# Chondroitinase ABC Promotes Axon Regeneration and Reduces Retrograde Apoptosis Signaling in Lamprey

**DOI:** 10.3389/fcell.2021.653638

**Published:** 2021-03-25

**Authors:** Jianli Hu, William Rodemer, Guixin Zhang, Li-Qing Jin, Shuxin Li, Michael E. Selzer

**Affiliations:** ^1^Shriners Hospitals Pediatric Research Center (Center for Neural Repair and Rehabilitation), Lewis Katz School of Medicine at Temple University, Philadelphia, PA, United States; ^2^Department of Anatomy and Cell Biology, Lewis Katz School of Medicine at Temple University, Philadelphia, PA, United States; ^3^Department of Neurology, Lewis Katz School of Medicine at Temple University, Philadelphia, PA, United States

**Keywords:** ChABC, neuronal death, axon regeneration, PTPσ, Akt, lamprey, SCI, FLICA

## Abstract

Paralysis following spinal cord injury (SCI) is due to failure of axonal regeneration. It is believed that axon growth is inhibited by the presence of several types of inhibitory molecules in central nervous system (CNS), including the chondroitin sulfate proteoglycans (CSPGs). Many studies have shown that digestion of CSPGs with chondroitinase ABC (ChABC) can enhance axon growth and functional recovery after SCI. However, due to the complexity of the mammalian CNS, it is still unclear whether this involves true regeneration or only collateral sprouting by uninjured axons, whether it affects the expression of CSPG receptors such as protein tyrosine phosphatase sigma (PTPσ), and whether it influences retrograde neuronal apoptosis after SCI. In the present study, we assessed the roles of CSPGs in the regeneration of spinal-projecting axons from brainstem neurons, and in the process of retrograde neuronal apoptosis. Using the fluorochrome-labeled inhibitor of caspase activity (FLICA) method, apoptotic signaling was seen primarily in those large, individually identified reticulospinal (RS) neurons that are known to be “bad-regenerators.” Compared to uninjured controls, the number of all RS neurons showing polycaspase activity increased significantly at 2, 4, 8, and 11 weeks post-transection (post-TX). ChABC application to a fresh TX site reduced the number of polycaspase-positive RS neurons at 2 and 11 weeks post-TX, and also reduced the number of active caspase 3-positive RS neurons at 4 weeks post-TX, which confirmed the beneficial role of ChABC treatment in retrograde apoptotic signaling. ChABC treatment also greatly promoted axonal regeneration at 10 weeks post-TX. Correspondingly, PTPσ mRNA expression was reduced in the perikaryon. Previously, PTPσ mRNA expression was shown to correlate with neuronal apoptotic signaling at 2 and 10 weeks post-TX. In the present study, this correlation persisted after ChABC treatment, which suggests that PTPσ may be involved more generally in signaling axotomy-induced retrograde neuronal apoptosis. Moreover, ChABC treatment caused Akt activation (pAkt-308) to be greatly enhanced in brain post-TX, which was further confirmed in individually identified RS neurons. Thus, CSPG digestion not only enhances axon regeneration after SCI, but also inhibits retrograde RS neuronal apoptosis signaling, possibly by reducing PTPσ expression and enhancing Akt activation.

## Introduction

Paralysis following spinal cord injury (SCI) is due to axon interruption and failure of regeneration. Accumulating evidence suggests that both extrinsic and intrinsic factors contribute to the inability of axons to regenerate. Among the key factors, chondroitin sulfate proteoglycans (CSPGs) are normal constituents of the perineuronal nets in central nervous system (CNS; Bruckner et al., 2000; Deepa et al., 2006), and are greatly elevated after SCI, both in rodents (Bradbury et al., 2002) and lampreys (Zhang et al., 2014). In the lamprey, CSPG levels peaked near the transection (TX) at 2 weeks post-TX, returning to normal by 10 weeks. *In vitro* experiments showed that CSPGs can inhibit neurite outgrowth (Snow et al., 1990; McKeon et al., 1991), while digestion of CSPGs with chondroitinase ABC (ChABC) can prevent macrophage-induced axon retraction (Busch et al., 2009). ChABC application *in vivo* leads to axon sprouting in the intact spinal cord (Galtrey et al., 2007). Moreover, intrathecal ChABC promoted growth of spinal axons and functional recovery in rats (Bradbury et al., 2002). Transgenic ChABC-mediated digestion of the CSPGs promoted growth of axons past a dorsal root crush (Cafferty et al., 2007), enhanced sensory recovery (Cafferty et al., 2008) and promoted compensatory sprouting and functional recovery after unilateral corticospinal tract (CST) lesion (Starkey et al., 2012). The molecular mechanisms for these effects are not certain, and the behavior of growth cones *in vitro* may not represent mechanisms of regeneration of mature axons *in vivo* (Jin et al., 2009). Although CSPGs might interfere with axon adhesion to extracellular matrix (Friedlander et al., 1994), the receptor-like protein tyrosine phosphatases (RPTPs), protein tyrosine phosphatase sigma (PTPσ) and leukocyte common antigen-related phosphatase (LAR), have been identified as transmembrane receptors for CSPGs (Shen et al., 2009; Fisher et al., 2011; Sharma et al., 2012). Genetic disruption of PTPσ promoted axon growth into CSPG-rich regions of SCI (Shen et al., 2009), and transgenic deletion of LAR increased growth of descending axons caudal to the lesion and enhanced locomotor recovery after SCI (Xu et al., 2015). This also was true for systemic injection of small peptide inhibitors of LAR (Fisher et al., 2011) and PTPσ (Lang et al., 2015). RPTPs exist in the plasma membrane as auto-inhibited dimers. When bound to ligands, they separate, triggering phosphatase activity (Hower et al., 2009). Similar to myelin-associated growth inhibitors such as Nogo (Monnier et al., 2003), CSPG-mediated inhibition of neurite growth appears to involve RhoA activation (Fisher et al., 2011).

Although removal of the polysaccharide side chains of CSPGs with ChABC enhances axon growth and functional recovery after SCI in mammalian partial injury models, it is not clear whether this involves true regeneration of injured axons, or to collateral sprouting by spared axons. Nor is it known how this treatment affects the expression of CSPG receptors and their downstream signaling pathways. Since lampreys have both CSPGs and their RPTPs, to get around the limitations of mammalian models, we used complete TX of lamprey spinal cord to determine whether these effects relate to true regeneration of lesioned axons.

In the lamprey, the main descending system that transmits commands from the brain to the spinal cord is composed of reticulospinal (RS) neurons, which are responsible for initiation of locomotion, steering, and equilibrium control (Deliagina et al., 2000). The 18 pairs of individually identified RS neurons have axons that extend the entire length of spinal cord and therefore, are always axotomized by a complete spinal cord TX. The perikarya of these identified RS neurons can be labeled retrogradely by application of a dye to the site of the fresh TX. In the lamprey, some spinal-projecting neurons are good regenerators and some are bad (Davis and McClellan, 1994; Jacobs et al., 1997). The latter often experience a very delayed form of apoptosis (Shifman et al., 2008; Busch and Morgan, 2012; Hu et al., 2013, 2017). In the present study, these features have been used to determine the effects of ChABC treatment on the regeneration of axons belonging to spinal-projecting neurons, on their retrograde death after spinal cord TX, and on a downstream pathway thought to participate in these effects. *In vitro* studies had indicated that Akt is an important downstream signaling molecule of CSPG receptors (Fisher et al., 2011). In addition, the deletion of phosphatase and tensin homolog (PTEN) has been reported to promote potent CNS axon regeneration after optic nerve injury (Park et al., 2008) and SCI (Liu et al., 2010; Du et al., 2015), and the effect of PTEN knockdown to promote survival of retinal ganglion cells (RGC) and regeneration of their axons appeared to involve activation of Akt (Yang et al., 2014; Guo et al., 2016). Therefore, we explored the role of Akt in mediating the axon regeneration and suppression of retrograde neuronal death produced by ChABC treatment after SCI in the lamprey.

## Materials and Methods

### Spinal Cord Transection, ChABC Treatment, and Retrograde Labeling

Wild-type larval lampreys, *Petromyzon marinus*, 10–14 cm in length (4–5 years old), were obtained from streams of Lake Michigan and maintained in fresh water tanks at room temperature (RT) until use. All animal procedures described in this report were performed with approval from the Temple University Institutional Animal Care and Use Committee (ACUP#: 4922). For spinal cord TX, animals were anesthetized by immersion in 0.1% tricaine methanesulfonate, and the spinal cord was exposed by an incision along the dorsal midline at the level of the fifth gill. TX of the spinal cord was performed with Castroviejo scissors. Completeness of TX was confirmed by retraction and visual inspection of the cut ends. For ChABC treatment, 1 μl ChABC (Cat# C2905, Sigma-Aldrich) dissolved in enzyme buffer was applied to the TX site and a pledget of Gelfoam soaked with 1 μl ChABC was placed gently on the surface of the spinal cord spanning the injury site. Control animals were treated with enzyme buffer. To label axon tips after SCI, we placed a pledget of Gelfoam soaked in 5% dextran tetramethylrhodamine (DTMR; Cat# D1817, Thermo Fisher Scientific) with enzyme buffer or ChABC into the TX gap. TXed lampreys recovered on ice for 2 h and then were returned to fresh water tanks at RT for 1, 2, 4, 8, and 11 weeks, at which times the brainstems were removed for fluorochrome-labeled inhibitor of caspase activity (FLICA) assay and then followed with PTPσ mRNA *in situ* hybridization (ISH). The spinal cords were carefully dissected out at 2 weeks and fixed with 4% paraformaldehyde (PFA) immediately. After washing with phosphate buffered saline (PBS), the axon tips were imaged with a fluorescence microscope. To assess axon regeneration at 10 weeks after the first TX at 5th gill, we performed the second TX at 5 mm caudal to the first TX and placed a pledget of Gelfoam soaked in 5% DTMR into the 2nd TX gap. The lampreys recovered on ice for 2 h and then were returned to fresh water tanks at RT for another 1 week to allow the DTMR to label the regenerated axons (Figures 2E–I). To retrogradely label the individual RS neurons for the convenience of pAkt-308 immunostaining analysis, 5% dextran Alexa Fluor 488 (DAF-488, Cat# D22910, Thermo Fisher Scientific) was placed into the TX gap. TXed lampreys recovered on ice for 2 h and then were returned to fresh water tanks at RT for 2, 4, or 8 weeks, at which times the brainstems were removed for pAkt-308 immunofluorescence staining.

### FLICA on Whole-Mounted Lamprey Brains

After the recovery times described above, animals were re-anesthetized by immersion in saturated benzocaine solution. Brains were dissected out in ice-cold lamprey Ringer’s buffer (110 mM NaCl, 2.1 mM KCl, 2.6 mM CaCl_2_, 1.8 mM MgCl_2_, and 10 mM Tris buffer; pH 7.4). The posterior and cerebrotectal commissures of the freshly dissected brains were split along the dorsal midline. Brains were incubated immediately at 4°C for 1 h in 150 μL 1 × FLICA labeling solution (Image-iT^TM^ Live Green Poly-Caspases Detection Kit, Cat# I35104, Molecular Probes; or Green Caspase-3 Staining Kit, Cat# PK-CA577-K183-25, PromoKine), which was diluted with PBS. Afterward, brains were washed five times with 1X wash buffer on a rotator at 4°C, 5 min per time. The alar plates of brains were deflected laterally and pinned flat to a small strip of Sylgard (Dow Corning Co., United States). The tissue was fixed in 4% PFA in PBS for 2 h at RT, and then washed three times in PBS at RT. Fluorescence images of brains were captured immediately with a Nikon 80i microscope. The whole procedure was conducted in the dark and all the samples were carefully protected from light. The brains were placed in 70% EtOH and kept at −20°C for PTPσ mRNA ISH. Control experiments were performed using brains from lampreys without spinal cord TX. All images were acquired using the same parameters.

### *In situ* Hybridization on Whole-Mounted Lamprey Brains After FLICA

*In situ* hybridization was carried out according to modifications of the chromogenic method previously described (Swain et al., 1994). The whole-mounted brains were placed in Eppendorf tubes and washed in PTW (0.1% Tween-20 in PBS), then pre-hybridized at ∼ 55°C in hybridization solution for 60 min (50% deionized formamide, 5X SSC, 100 mg/ml Torula yeast RNA, 100 mg/ml wheat germ tRNA, 50 mg/ml heparin, 0.1% Tween-20). Hybridization was carried out by adding digoxin-labeled PTPσ antisense RNA probes, 1 μg/ml in hybridization solution, to the brain samples, which were kept at ∼ 55°C on a rotator overnight. Brains were washed in hybridization solution at 55°C followed by washes in PTW and PBT (0.1% bovine serum albumin, 0.2% Triton X-100 in PBS) at RT. Anti-Digoxigenin-AP Fab fragments (Cat# 11093274910, Roche Applied Science) were applied 1:1,000 to the brain samples at 4°C on a rotator overnight. The samples were washed sequentially in PBT and SMT (100 mM NaCl, 50 mM MgCl2, 100 mM Tris, pH 9.5, 0.1% Tween-20). The chromogenic reaction was carried out in a solution containing 20 μl of NBT/BCIP stock solution (Cat# 11681451001, Roche Applied Science) in each 1 ml of SMT on ice in the dark for 1 h, or until the reaction was completed, as determined by monitoring under a dissecting microscope. Finally, brain samples were washed in PBS, and the meninges were stripped gently from the posterior surface of the brain, using a forceps under a dissecting microscope. The brains were mounted onto glass slides and bright-field images were captured with a Nikon 80i microscope.

### Calculation of Probabilities of FLICA- and PTPσ-Positive Identified RS Neurons

The RS neurons were identified individually in brain wholemounts labeled retrogradely with DTMR, based on their characteristic morphologies, sizes, and locations (Jacobs et al., 1997). Thus far, there are no molecular markers specific for individual RS neurons, although retrograde labeling combined with immunohistochemistry for developmentally regulated genes has been used to study the segmental development of the embryonic lamprey hindbrain (Murakami et al., 2004). The number of PTPσ-positive neurons were counted separately for each of the individually identified RS neurons in each brain. Then, for each of the individually identified RS neurons, the number of PTPσ-positive neurons was divided by the total number of neurons (2) of that individual type (PTPσ-positive and -negative) in each brain, and the percentages were considered the probability of PTPσ positivity for each of the identified RS neurons. For example, among the five brains treated with ChABC and surveyed at 2 weeks post-TX, three M1 neurons were PTPσ positive. Since each brain has two M1 neurons, the total number of M1 neurons was 2 × 5 = 10. Thus, the probability of PTPσ positivity for the M1 neuron in this ChABC-treated group was (3÷10) × 100 = 30%. This method also was used to calculate the percent of FLICA-positive identified RS neurons.

### Western Blotting

The brains were collected from lampreys under a dissecting microscope. To investigate the expression of Akt in brains (with or without ChABC treatment), we dissected out the brain from the olfactory lobe to the obex. The tissues were snap-frozen in liquid nitrogen and homogenized in cold lysis buffer (Cat# C3228, Sigma-Aldrich) supplemented with 1× protease inhibitor cocktail (Cat# P8341, Sigma-Aldrich). After brief centrifugation to remove debris, the total protein concentration in supernatants was determined, using Bio-Rad (Hercules, CA, United States) DC protein assay reagents (Cat# 500-0006, Bio-Rad). After 10 min of heating at 75°C in loading buffer (Cat# NP 0007, Invitrogen) supplemented with reducing reagent (Cat# NP 0004, Invitrogen), 25 μg of protein were loaded from each sample. The protein was separated in 4–12% NuPAGE^®^ Bis-Tris gradient mini gels (Cat# NP 0321BOX, Invitrogen), and transferred onto a PVDF membrane, using a Bio-Rad transblot apparatus. The membranes were blocked in 5% non-fat dry milk in TRIS-buffered saline (TBS) for 1 h at RT. Membranes were probed with an anti-Akt-T308 antibody (Cat# 2965, Cell Signaling) diluted 1:1000 at RT for 1 h, or anti-Actin (Cat# MAB1501, Chemicon) diluted 1:10,000 at 4°C overnight. After washes with TBS, the blots were incubated with secondary antibodies IRDye 800CW goat anti-rabbit IgG (Cat# 926-32211, LI-COR) or IRDye 680RD goat anti-mouse IgG (Cat# 926-68070, LI-COR) at 1: 20,000 for 1 h at RT in the dark. The blots were washed three times with TBS, 10 min each, scanned and quantified with an Odyssey CLx (LI-COR), and processed with Adobe Photoshop (San Jose, CA, United States).

### Immunohistochemistry

To test whether *in vivo* ChABC treatment can digest CSPGs in lamprey spinal cord. We applied ChABC onto the intact spinal cord at the level of the fifth gill, and sacrificed the lampreys after 4 h recovery. We also applied ChABC *in vivo* immediately after TX at the fifth gill, and allowed the lampreys to survive for 2 weeks before being sacrificed. The spinal cords between the second gill and 5 mm caudal to the seventh gill were fixed, dehydrated and embedded in paraffin. Sagittal 10 μm thick sections were mounted onto glass slides. After de-paraffinization and rehydration, sections underwent the chromogen reaction. Endogenous horseradish peroxidase (HRP) was quenched with 5% H_2_O_2_ in methanol for 30 min at RT. Sections then were washed three times with PBS, 10 min each. All the sections were incubated with 10% fetal bovine serum (FBS)/0.2% Tween-20/PBS for 1 h at RT and then incubated with primary antibody 2B6 (Cat# 270432, Seikagaku Biobusiness Corporation) at a dilution of 1:200 in 10% FBS/0.2% Tween-20/PBS overnight at 4°C. Sections were washed three times with PBS, 10 min each, and then incubated with either goat anti-mouse IgG-conjugated HRP (Cat# SC-2005, Santa Cruz) at 1:200 or donkey anti-mouse IgG-conjugated Alexa Fluor^®^ 594 (Cat# R37121, Thermo Fisher Scientific) at 1:200, in 10% FBS/0.2% Tween-20/PBS for 1 h at RT. All sections were washed three times with PBS, 10 min each. After incubation in HRP-secondary antibody, sections were washed with PBS and the chromogen reaction was performed with diamino benzidine (DAB). If sections were incubated in fluorescently tagged secondary antibody, the sections were washed with PBS and mounted with Fluoromount-G (Cat# 0100-01, SouthernBiotech). Brightfield or fluorescence images were captured with a Nikon 80i microscope.

To measure levels of Akt phosphorylation at threonine 308 in individual identified neurons, after fixation, dehydration, and paraffin embedding, 10 μm thick paraffin sections were mounted onto glass slides for further investigation. All of the brain sections were de-paraffinized, rehydrated, and washed in PBS. Antigen retrieval was performed as follows: sections were immersed in the sodium citrate buffer (10 mM sodium citrate, pH 6.0). The buffer was boiled for 20 min, and the sections allowed to cool for 20 min at RT. Sections were rinsed in PBS twice for 5 min each time. All the sections were blocked with 10% FBS/0.2% Tween-20/PBS for 1 h at RT, and incubated overnight with primary antibody anti-Akt-T308 (Cat# 2965, Cell Signaling) at 1:1000 in 10% FBS/0.2% Tween-20/PBS at 4°C. Sections were washed three times with PBS, 10 min each and then incubated with donkey anti-rabbit Alexa Fluor^®^ 594 (Cat# A21207, Thermo Fisher Scientific) at 1:200, in blocking buffer for 1 h at RT. After incubation in secondary antibody, sections were washed three times with PBS, 10 min each time. Autofluorescence was carefully quenched with the TrueView kit (Cat# SP-8400, Vector TrueView). Sections were mounted with Fluoromount-G (Cat# 0100-01, SouthernBiotech). DAF-488 and pAkt-308 fluorescence signaling were captured with a Nikon 80i microscope under consistent parameters to allow quantification of pAkt-308 fluorescence. All brain sections with identified neurons were collected and quantified with NIS-Elements AR 3.10. For each brain section, all the identified neurons that were filled with DAF-488 were outlined and pAkt-308 intensity was measured. Background fluorescence intensity was measured by outlining the area adjacent to the brain. The fluorescence intensity for each section was calculated as follows: the background fluorescence intensity was subtracted from the mean fluorescence intensity within identified neurons in the same section. For each animal, the average fluorescence intensity from all the sections was calculated. Then an overall mean fluorescence was calculated as the mean of all these average intensities.

### Statistical Analysis

Data sets were analyzed with InStat software (GraphPad), normally distributed data were further analyzed by InStat to determine if standard deviations (SD) were equal. An unpaired *t*-test was used for comparison between data sets with equal SD. For western blots, to avoid between-blot variation, all the groups were normalized against loading controls (actin). Then the experimental groups were compared with their respective normalized control groups, whose relative densities were assigned a value of 1. A paired *t*-test was performed to compare the density difference between groups. The effects of ChABC on apoptosis signaling was determined for individual identified neurons, by comparing FLICA labeling for the same RS neurons in control and ChABC-treated groups, using the paired *t*-test. For normally distributed data sets requiring multiple group comparisons, we used one-way analysis of variance (ANOVA) followed by the Tukey’s multiple comparisons test. For correlation analysis, we used the Pearson correlation test. All values were expressed as mean ± SEM.

## Results

### ChABC Digests CSPGs in Lamprey Spinal Cord

**FIGURE 1:**
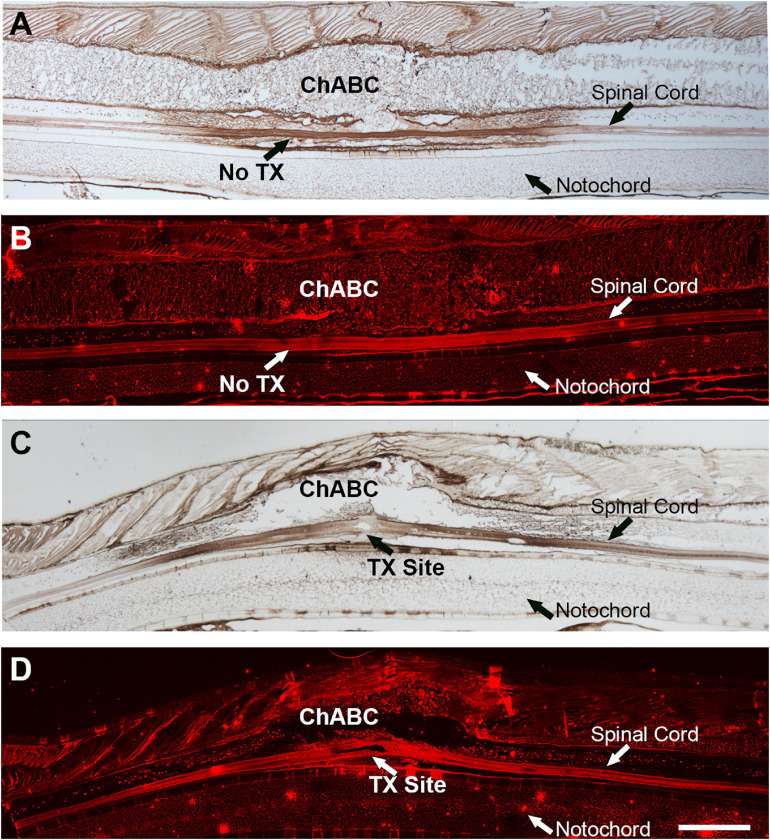
ChABC treatment of lamprey spinal cord after transection. Stumps of digested CSPGs were labeled with mAb 2B6 and imaged in sagittal sections of spinal cord by colorimetric immunohistochemistry **(A,C)** or Alexa Fluor-594 immunofluorescence **(B,D)**. **A**,**B** are intact lamprey spinal cord treated with ChABC. **C,D** are sections of a spinal cord that had been transected and treated with ChABC 2 weeks previously. Scale: 1 mm.

Chondroitinase ABC was applied *in vivo* onto the lamprey spinal cord at the level of the 5th gill, with or without TX (detail in section “Materials and Methods”). After ChABC application, there was strong staining of CSPG stumps (2B6) in control and TXed spinal cords (Figures 1A,C), suggesting that ChABC had digested the sugar chains of CSPGs, and thereby exposed the stumps (2B6). H_2_O_2_ was used to eradicate endogenous HRP activity during IHC staining and chromogenic reaction. The results were confirmed by immunofluorescence staining with the 2B6 antibody, to show the distribution of CSPG stumps after ChABC treatment in control and TXed spinal cords. Figures 1B,D show strong immunofluorescence in the same area as indicated in Figures 1A,C. These results confirmed that ChABC application *in vivo* can digest endogenous CSPGs effectively in lamprey CNS, either in control lamprey, or after spinal cord TX.

### Digestion of CSPGs With ChABC at the Site of Injury Inhibits Axon Retraction in the Proximal (Rostral) Stump at Early Stage and Promotes Axon Regeneration at Late Stage

**FIGURE 2:**
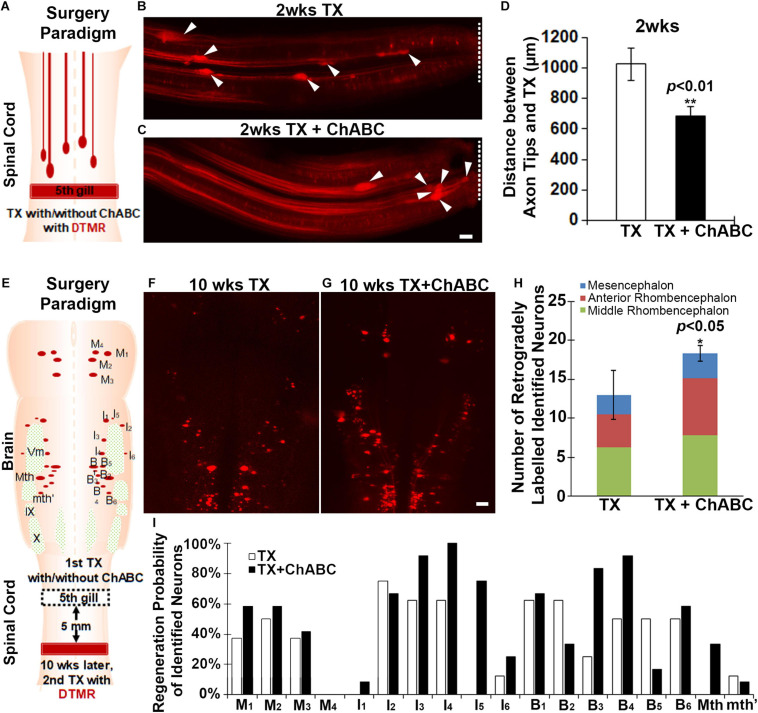
ChABC slows initial axon retraction *in vivo* at 2 weeks and promotes axon regeneration *in vivo* at 10 weeks post-TX. **(A)** Experimental design for detecting the axon tips at 2 weeks post-TX. A solution of DTMR and either ChABC or control enzyme buffer was applied to a fresh spinal cord TX. **(B,C)** Axon tips (arrowheads) in the lamprey spinal cord back-filled with DTMR at 2 weeks post-TX (TX sites are labeled by dashed lines). **(D)** Mean distances of axon tips from the center of the TX site (dashed line). **p < 0.01, *n* = number of axon tips. **(E)** Experimental design for detecting the neurons whose axons have regenerated by 10 weeks post-TX. The TX site was treated with either control enzyme buffer **(F)**, or ChABC **(G)** at the time of injury. Ten weeks later, DTMR was applied to a second TX 5 mm caudal to the first, and 1 week allowed for retrograde transport of the dye. **(H)** A graph showing an increased number of retrogradely labeled neurons in brains of animals treated with ChABC compared to controls. **(I)** The probability for each identified neuron that its axon will have regenerated at 10 weeks post-TX. *p < 0.05, *n* = 5 lampreys per group. Error bars: SEM. Scale bar: 100 μm.

At the early stage after SCI in the lamprey, transected axons can form axon tips which undergo retraction first and then begin to grow forward toward the TX site, reaching the injury scar by 4 weeks. These axon tips can be imaged *in vivo*, and thereby can be assessed by measuring the distance between the axon tips and TX site (Jin et al., 2009; Hu et al., 2017). We transected the spinal cords at the level of the 5th gill and applied either enzyme buffer or ChABC with DTMR to the TX site for 2 weeks (Figure 2A). The whole-mounted free spinal cords were carefully dissected out. The images of axon tips were taken by fluorescence microscopy, so that the distances between individual axon tips (arrowheads in Figures 2B,C) and the TX sites (dashed lines in Figures 2B,2C) could be measured. The mean distances between the axon tips and TX sites in ChABC-treated spinal cords (683 ± 62 μm; *n* = 30 from five lampreys) were shorter than that in enzyme buffer-treated ones (1026.03 ± 106 μm; *n* = 31 from five lampreys, *p* < 0.01) at 2 weeks post-TX (Figure 2D). This result suggested that ChABC digestion of CSPGs inhibits the retraction of transected axons, either by reducing the retraction distance or by promoting the early start of axon regrowth.

We also assessed the axon regeneration after ChABC treatment at late stage (10 weeks post-TX). A first TX was performed at the fifth gill and Gelfoam was applied with either enzyme buffer (control) or ChABC in enzyme buffer. After 10 weeks, a 2nd TX was performed 5 mm caudal to the first TX, and DTMR applied as a retrograde tracer to both groups (control vs. ChABC) (Figure 2E). After 1 more week, the retrogradely labeled RS neurons in the brain were counted carefully and these were considered neurons whose axons had undergone true regeneration (not collateral sprouting) at least 5 mm beyond the TX (Figure 2E). The total number of DTMR-positive identified RS neurons was greatly increased at 10 weeks post-TX with ChABC treatment compared to control enzyme buffer treatment (Figures 2F–H). We organized all of the identified RS neurons by their locations in brain (M_1_–M_4_ are in mesencephalon; I_1–_I_6_ are in anterior rhombencephalon; B_1–_B_6_, Mth and mth′ are in the middle rhombencephalon). There were increased numbers of DTMR-labeled identified RS neurons in all three of these regions, among which the RS neurons in the anterior rhombencephalic region showed the most dramatic regeneration (Figure 2H). Since individual identified RS neurons have different regeneration probabilities, the effects of ChABC treatment might be different for each of the individual identified RS neurons. We calculated the regeneration probability of individual identified RS neurons after TX with or without ChABC treatment (Figure 2I). Except for M_4_, I_2_, B_2_, B_5_, and mth′, all the other identified RS neurons benefited from ChABC treatment. The poorly regenerating neurons I_1_, Mth, B_3_, and B_4_ and the good regenerators M_1_, I_3_, I_4_, and I_5_, all had greater regeneration probabilities with ChABC treatment. Thus, in lamprey, ChABC promotes true axon regeneration after spinal cord TX.

### Digestion of CSPGs With ChABC Reduces Retrograde Neuronal Apoptosis Signaling

**FIGURE 3:**
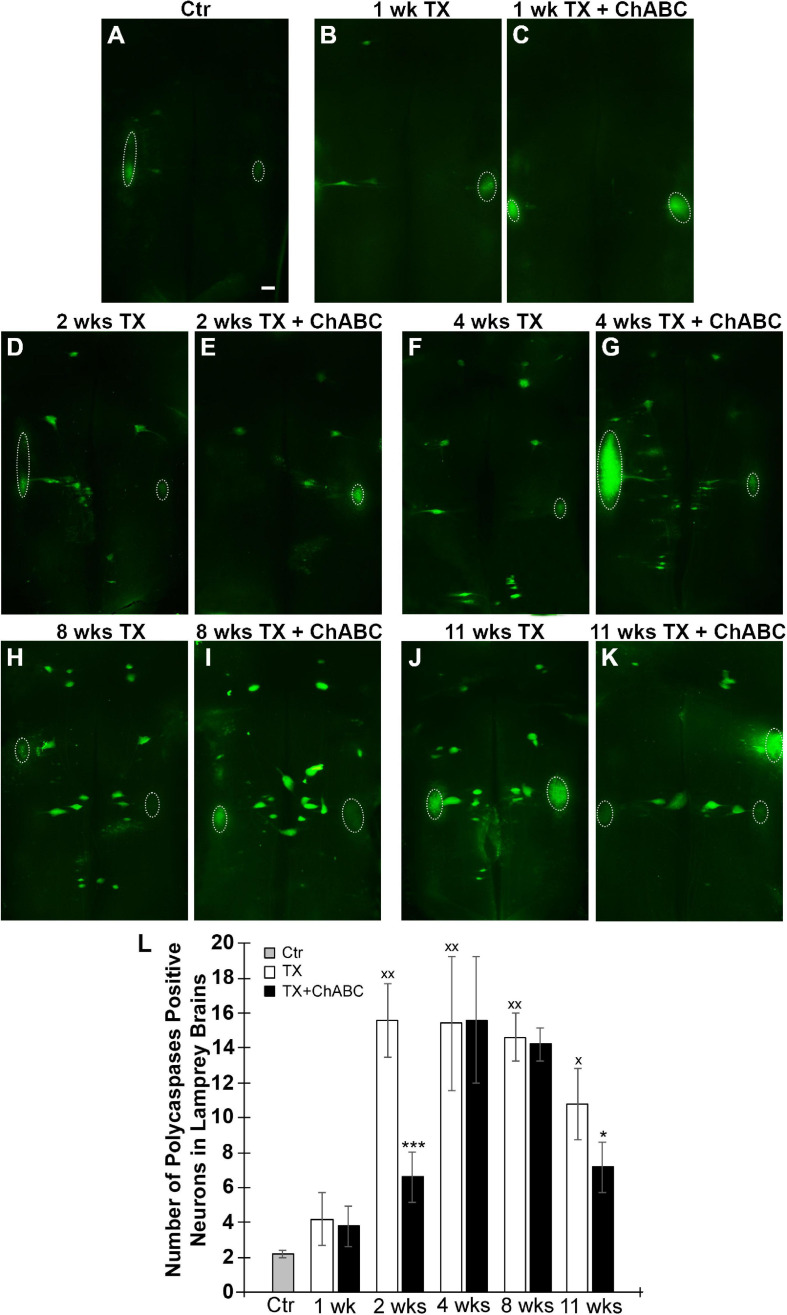
Polycaspase activation in RS neurons is suppressed by ChABC. Spinal cords were transected at the 5th gill with/without ChABC, and allowed to recover for 1–11 weeks. **(A)** FLICA on control brain (without TX). ChABC did not affect the number of polycaspase positive (polycaspase FLICA) RS neurons at 1 week post-TX (**B** vs. **C**), but reduced it significantly at 2 weeks (**D** vs. **E**) and at 11 weeks (**J** vs. **K**, *p < 0.05), but not at 4 (**F** vs. **G**) or 8 weeks (**H** vs. **I**). **(L)** A graph showing the number of polycaspase positive identified RS neurons per brain at 1, 2, 4, 8, and 11 weeks post-TX with or without ChABC. Polycaspase positive RS neurons increased greatly at 2, 4, 8, and 11 weeks comparing to control brain (^*x*^*p* < 0.05, ^*xx*^*p* < 0.01); ChABC reduced polycaspase positive RS neurons at 2 (***p < 0.001) and 11 weeks (*p < 0.05). The large fluorescent accumulations (outlined by circles) seen laterally are out of focus cranial motor nuclei, which are axotomized close to their perikaryal at the time of live dissection, so their neurons rapidly turn caspase-positive. *n* = 5 lampreys per group. Error bars: SEM. Scale bar: 200 μm.

**FIGURE 4:**
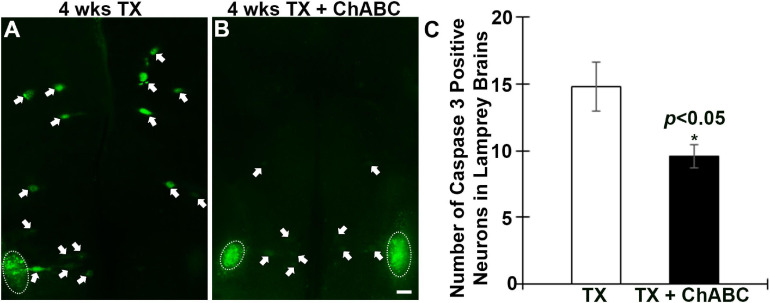
Active caspase 3 in RS neurons is reduced by ChABC at 4 weeks post-TX. Spinal cords were transected at the 5th gill with or without ChABC, and allowed to recover for 4 weeks. ChABC reduced the number of caspase 3-positive RS neurons at 4 week post-TX significantly (**A** vs. **B**). **(C)** A graph showing the number of caspase 3-positive identified RS neurons per brain at 4 weeks post-TX with or without ChABC. Arrows point to the active caspase 3 positive RS neurons, and circles outline cranial motor nuclei, whose neurons rapidly become caspase positive when they are axotomized during brain dissection. *p < 0.05, *n* = 5 lampreys per group. Error bars: SEM. Scale bar: 100 μm.

Fluorochrome-labeled inhibitor of caspase activity staining was performed on brains at 1, 2, 4, 8, and 11 weeks after spinal cord TX. Compared to uninjured controls (Figure 3A), the number of all RS neurons showing polycaspases activity increased significantly at 2, 4, 8, and 11 weeks post-TX (Figures 3D,F,H,J,L). ChABC did not affect the number of polycaspases positive (FLICA+) RS neurons at 1 week post-TX (Figure 3B vs. C, Figure 3L), but reduced it significantly at 2 weeks (Figure 3D vs. E, Figure 3L). This reduction effect disappeared at 4 weeks (Figure 3F vs. G, Figure 3L) and 8 weeks (Figure 3H vs. I, Figure 3L). Interestingly, at 11 weeks after TX, the number of FLICA+ neurons decreased again in ChABC-treated lampreys (Figure 3J vs. K, Figure 3L), suggesting that the ChABC effects on retrograde neuronal death outlast the caspase activation. This is consistent with the effects of ChABC to increase axon regeneration at 10 weeks post-TX (Figures 2E–I). It has been reported that caspases are not involved only in apoptosis, but also play critical roles in multiple cellular processes in the nervous system (Hu et al., 2013). The polycaspases FLICA reagent that we used in this study labels all active caspases and does not specifically target any single caspase. This may explain why ChABC did not affect the number of poly-caspase positive neurons at 4 and 8 weeks post-TX. To clear up this ambiguity, caspase 3-specific FLICA was used (Figure 4), which targets only neurons undergoing apoptosis. ChABC significantly reduced the number of caspase 3-positive RS neurons at 4 weeks post-TX (Figure 4). Thus ChABC treatment had a beneficial effect by reducing retrograde apoptosis after SCI.

### ChABC Inhibits PTPσ mRNA Expression

**FIGURE 5:**
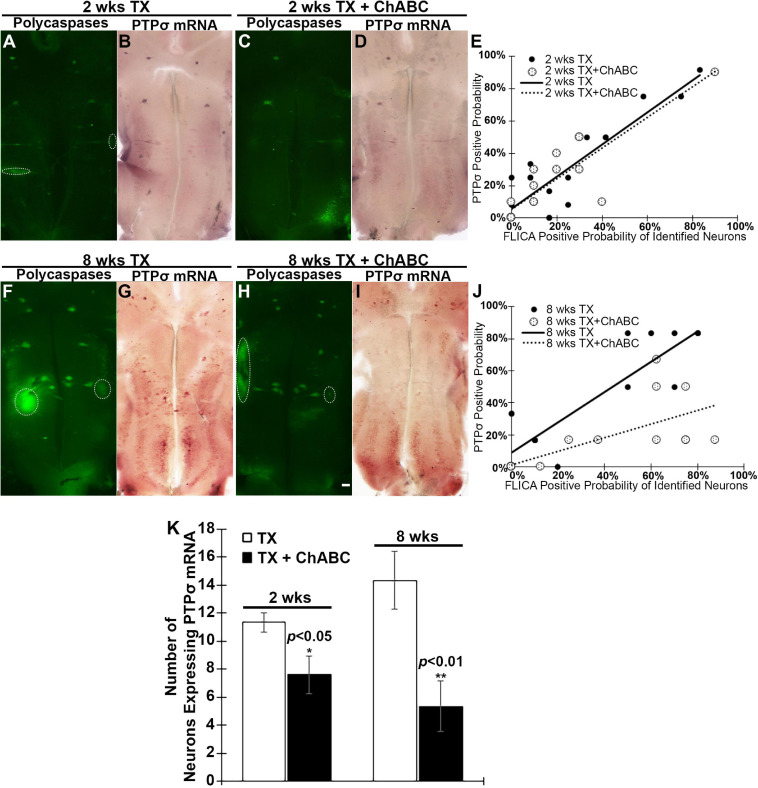
ChABC reduces PTPσ mRNA expression in brain at 2 and 8 weeks post-transection. At 2 weeks post-TX, ChABC reduced caspase activation (**A** vs. **C**), and also reduced the number of neurons expressing PTPσ mRNA (**B** vs. **D**). **(E)** PTPσ mRNA expression correlated strongly with FLICA in RS neurons at 2 weeks post-TX, both with control enzyme buffer treatment (*r* = 0.8886, *p* < 0.001) and with ChABC (*r* = 0.8729, *p* < 0.001). The effect of ChABC on caspase activity disappeared at 8 weeks post-TX (**F** vs. **H**), but the number of neurons expressing PTPσ mRNA was still reduced (**G** vs. **I**). **(J)** At 8 weeks post-TX, there was a strong correlation between PTPσ mRNA and FLICA in identified RS neurons, both with control enzyme buffer (*r* = 0.8985, *p* < 0.001) and with ChABC treatment (*r* = 0.7145, *p* < 0.001). **(K)** The number of identified RS neurons expressing PTPσ mRNA was reduced greatly by ChABC treatment at 2 and 8 weeks post-TX. Circles outline cranial motor nuclei, whose neurons rapidly become caspase positive when they are axotomized during brain dissection. *p < 0.05, **p < 0.01, *n* = 5 lampreys per group. Error bars: SEM. Scale bar: 200 μm.

Because PTPσ is thought to be a receptor for CSPGs, we determined the effect of digesting CSPGs with ChABC on the expression of PTPσ. ISH was used to detect the level of PTPσ mRNA expression in identified RS neurons at 2 and 8 weeks post-TX. In this set of experiments, we identified the 18 pairs of RS neurons by their specific anatomical locations and unique cell body morphologies. We found that ChABC not only reduced polycaspases activation at 2 weeks post-TX (Figure 5A vs. C), but also reduced the number of neurons expressing PTPσ mRNA (Figure 5B vs. D, Figure 5K). PTPσ mRNA expression correlated with polycaspases FLICA labeling in RS neurons at 2 weeks post-TX, both without (*r* = 0.8886, *p* < 0.001) and with ChABC treatment (*r* = 0.8729, *p* < 0.001) (Figure 5E). Although the effect of ChABC on polycaspases activity had disappeared by 8 weeks post-TX (Figure 5F vs. H), the number of neurons expressing PTPσ mRNA remained reduced (Figure 5G vs. I, Figure 5K). The correlations persisted at 8 weeks post-TX (Figure 5J; *r* = 0.8985, *p* < 0.0001, and *r* = 0.7145, *p* < 0.001, respectively).

### ChABC Increases Akt Activation

**FIGURE 6:**
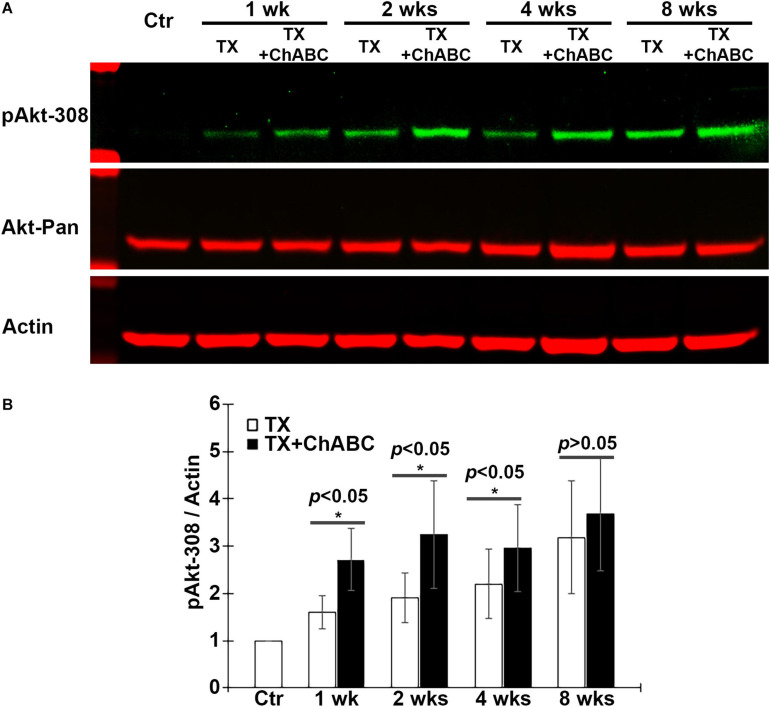
ChABC increases Akt phosphorylation at Threonine 308 (T308) in brain post-TX. **(A)** Brain homogenates from control lampreys and at 1, 2, 4, and 8 weeks post-TX, treated with ChABC or control enzyme buffer, were examined by western blots and probed with mAbs against pAkt-308, total Akt (Akt-pan) and actin as a loading control. **(B)** At each time point, ChABC treatment produced a significant increase in pAkt-308 compared with control enzyme buffer (*p < 0.05, *n* = 5 lampreys per group). Error bars: SEM.

Akt is an important downstream target signaling molecule for CSPG receptors in neurons *in vitro* (Fisher et al., 2011), and Akt activity is thought to be a signaling molecule that promotes axon regeneration after SCI. Therefore, the effect of ChABC on the activation status of Akt was investigated, using phosphorylation at threonine 308 (pAkt-308) as an indicator. ChABC treatment after SCI increased Akt phosphorylation levels in the brain at 1, 2, 4, and 8 weeks post-TX (Figures 6A,B). This long-term activation of Akt after ChABC treatment is consistent with its enhancement of long-term axon regeneration described above (Figures 2E–I).

**FIGURE 7:**
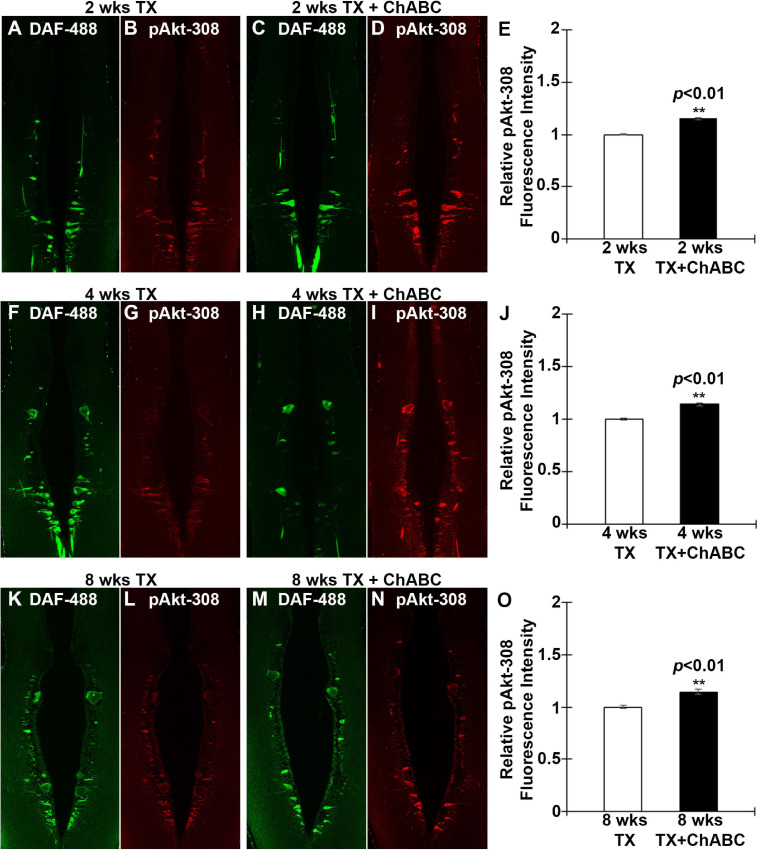
ChABC treatment increases the axotomy-induced activation of Akt in identified RS neurons. RS neurons were retrogradely labeled with DAF-488 and treated with ChABC or control enzyme buffer. Horizontal paraffin sections were prepared and imaged at 2 weeks post-TX (green, **A** and **C**). Sections were immunostained for pAkt-308 (Red, **B** and **D**). There was a small but statistically significant increase in pAkt-308 fluorescence intensity in identified RS neurons of the ChABC-treated group, relative to the control buffer-treated animals, whose mean intensity was defined as 1 (**E**; **p < 0.01, *n* = 3 lampreys per group). This also was true at 4 weeks (**F–J**; **p < 0.01, *n* = 5 lampreys per group) and at 8 weeks post-TX (**K–O**; **p < 0.01, *n* = 3 lampreys per group). Error bars: SEM. Scale bar: 100 μm.

Since Akt is expressed widely in both neurons and glial cells, the western blots cannot specify whether Akt activation occurs specifically in axotomized neurons (Figure 6). Therefore, we determined the activation status of Akt in individually identified RS neurons after ChABC treatment. RS neurons were retrogradely labeled by DAF-488 applied to an acute spinal cord TX, with or without ChABC treatment. The glial cells would not be labeled because they lack the long axons that would allow for retrograde labeling. Lampreys were sacrificed at 2, 4, and 8 weeks post-TX, and the brains fixed and processed for paraffin sectioning. The expression levels of pAkt-308 in the individual RS neurons were determined by immunofluorescence staining. The individual identified neurons were recognized with the retrogradely labeled DAF-488 (green, Figures 7A,C,F,H,K,M), and then by pAkt-308 immunofluorescence (red, Figures 7B,D,G,I,L,N). Akt phosphorylation intensity was quantified in the DAF-488 retrogradely labeled neurons. Thus, we included only neurons that were filled with the retrograde dye DAF-488, i.e., RS neurons. At 2, 4, and 8 weeks post-TX, the fluorescence intensity in identified RS neurons of ChABC-treated lampreys was approximately 15% greater than in those treated with control enzyme buffer (Figures 7A–E, F–J, and K–O), respectively; *p* < 0.01). Thus digestion of CSPGs with ChABC significantly increased the level of activation of the pro-growth signaling molecule Akt in the individually identified RS neurons of the lamprey brain.

## Discussion

We investigated the effects of removing the polysaccharide side chains of CSPGs with ChABC on axon regeneration of RS neurons, and on retrograde apoptosis of their cell bodies in the brainstem, after SCI in the lamprey. IHC of CSPG stubs confirmed the digestion of CSPGs by application of ChABC *in vivo*. FLICA labeling was used to quantify caspase activation, i.e., apoptosis signaling, which previously was shown to be increased after spinal cord TX, primarily in “bad-regenerating” identified RS neurons in lamprey brain (Barreiro-Iglesias and Shifman, 2012; Hu et al., 2013).

### Digestion of CSPGs With ChABC Reduces Retrograde Apoptotic Signaling

Caspases are a family of cysteine proteases that are found in a wide range of animals, from worms to humans, and are involved in apoptosis. The poly-caspase FLICA we used in the present study detects most caspases, including caspase 1, 3, 4, 5, 6, 7, 8, and 9. The number of identified RS neurons containing activated polycaspases increased significantly at 2, 4, and 8 weeks post-TX, compared to controls. These findings are consistent with our previous findings that neurons known to be bad regenerators eventually die by a very delayed form of TUNEL-positive apoptosis (Shifman et al., 2008). CSPGs, which are normal constituents of the perineuronal nets in CNS (Bruckner et al., 2000; Deepa et al., 2006), are greatly elevated after SCI (Bradbury et al., 2002; Zhang et al., 2014). We successfully digested the elevated CSPGs by ChABC at the TX site in the spinal cord. Digestion of CSPGs significantly reduced the number of polycaspase positive RS neurons at 2 and 11 weeks post-TX, but showed no beneficial effects at 4 and 8 weeks post-TX. However, because in addition to their role in apoptosis, many caspases also play critical roles in multiple physiological processes in the nervous system, such as dendritic remodeling (Kuo et al., 2006; Williams et al., 2006) and synaptic plasticity (Lu et al., 2006; Li et al., 2010). Even caspase-3 has been found in some cases to participate in non-apoptotic functions in CNS (D’Amelio et al., 2010), but caspase-3 is an executioner caspase, which is more directly involved in apoptosis than are the upstream caspases. Therefore, we used caspase 3-specific FLICA to selectively target RS neurons undergoing apoptosis. ChABC significantly reduced the number of those RS neurons at 4 weeks post-TX. Based on the caspase 3 results at 4 weeks, it is reasonable to conclude that lack of reduction in polycaspace activity does not exclude reduced caspase 3 activity after ChABC treatment at 8 weeks, as well. We could have repeated the caspase 3-specific assays at 8 weeks, but felt that the 4-week result was proof of principle. Thus, ChABC treatment after SCI can greatly decrease retrograde apoptotic signaling.

### Digestion of CSPGs With ChABC Promotes Axon Regeneration

Chondroitinase ABC treatment has beneficial effects over the transected axons. At 2 weeks post-TX, ChABC treatment reduced the distance between the axon tips and TX sites. Moreover, it greatly increased the number of individually identified RS neurons that could be labeled retrogradely from 5 mm caudal to a spinal cord TX at 10 weeks post-TX, indicating that ChABC treatment promotes axon regeneration after SCI. The beneficial effects of ChABC on identified RS neurons were seen in the mesencephalon, and the anterior and middle rhombencephalon. The ChABC treatment also changed the probabilities of regenerating for individual identified RS neurons, which included both bad regenerators and good regenerators. These findings suggest that ChABC treatment is generally beneficial for axon regeneration of these identified RS neurons. This might be due to retrograde signaling in the injured axons due to their interaction with CSPGs secreted at the site of injury. Previous reports in mammals suggested that *in vivo*, ChABC application in the intact spinal cord can induce axon sprouting (Galtrey et al., 2007). In rats with bilateral dorsal column lesions, ChABC treatment promoted growth of spinal axons and functional recovery (Bradbury et al., 2002). Digestion of the CSPGs with ChABC enhanced sensory recovery after unilateral cervical rhizotomy at C5, C6, C8, and T1, sparing C7. This was accomplished via reorganization of intact C7 primary afferent terminals – not by regeneration of severed afferents back into the spinal cord (Cafferty et al., 2008). Unilateral pyramidotomy in spinal cord-injured mice elicited robust sprouting of the uninjured CST, with numerous axons observed crossing the midline in the brainstem and spinal cord, and terminating in denervated gray matter. This was accompanied by restoration of function (Starkey et al., 2012). Taken together, these studies suggest that digestion of CSPGs with ChABC enhances axon sprouting and functional recovery after SCI in mammalian models, but because these models involved partial SCI, it is unclear whether this beneficial effect was due entirely to collateral sprouting by spared axons, or also involves true regeneration of injured axons. This ambiguity was eliminated in the current study on lampreys, because we performed complete spinal cord TX. Thus the increased growth of RS axons beyond the lesion was due to true regeneration, and could not be accounted for by compensatory collateral sprouting by spared axons.

### Role of PTPσ in Retrograde Neuronal Death

Chondroitinase ABC treatment at the time of SCI significantly inhibited PTPσ mRNA expression in the perikarya of the axotomized identified RS neurons, as assessed at 2 and 8 weeks post-TX. This was accompanied by a concomitant reduction in retrograde neuronal apoptotic signaling (poly-caspase FLICA), and is consistent with a role for PTPσ in retrograde neuronal apoptosis. This was suggested previously by the selective expression of PTPσ in “bad regenerator, bad survivor” RS neurons, which became FLICA-positive after SCI (Hu et al., 2013). More intriguingly, Akt activation (pAkt-308) was enhanced in brains after ChABC treatment, as determined by western blotting, and confirmed in individual identified RS neurons by immunostaining.

### Role of PTPσ in Axon Regeneration

Two RPTPs, PTPσ, and LAR, have been identified as transmembrane receptors for CSPGs (Shen et al., 2009; Fisher et al., 2011; Sharma et al., 2012). Genetic disruption of PTPσ promoted axon growth into CSPG-rich regions of SCI (Shen et al., 2009). Transgenic deletion of LAR increased growth of descending axons caudal to the lesion and enhanced locomotor recovery after SCI (Xu et al., 2015). This also was true for systemic injection of small peptide inhibitors of LAR (Fisher et al., 2011) and PTPσ (Lang et al., 2015). Our group used antisense morpholinos (MOs) to knock down lamprey PTPσ *in vivo* and studied its direct effects on the axon regeneration and retrograde neuronal death after SCI. Unexpectedly, we found that PTPσ knockdown in lamprey reduced axon regeneration and neuronal survival beginning between 10 and 20 weeks after TX (Rodemer et al., 2020). Those results seem to be inconsistent with the putative role of PTPσ in mammalian axon regeneration (Lang et al., 2015), and with the correlation between PTPσ mRNA and post-TX retrograde apoptotic signaling seen even after ChABC treatment in current study. In our previous report, the lack of activated caspases in RS neurons, and the long latency after PTPσ knockdown *in vivo* indicated that enhanced supraspinal neuronal death might result from non-apoptotic mechanisms: by incidentally transfected infiltrating immune cells, or trophic deprivation, or autonomous autophagic mechanisms (Rodemer et al., 2020). It also is possible that the redundancy of CSPG receptors mitigated the beneficial effect of *in vivo* PTPσ knockdown. The use of ChABC can avoid the complications or concerns raised with PTPσ knockdown *in vivo.* The ChABC-induced digestion of elevated CSPGs around the TX site restores the CSPG levels in the environment of the injury site to those found in the un-injured spinal cord. This manipulation is easy to perform and does not directly interfere with other physiologically critical molecules *in vivo*. Thus, ChABC digestion of elevated CSPGs may be beneficial to axon regeneration and neuronal survival in more than one mechanism.

### Digestion of CSPGs With ChABC Reduces Expression of PTPσ

Previously, we reported that in lamprey CNS, CSPGs were widely distributed in the extracellular matrix, as well as in cell bodies of the gray matter (Zhang et al., 2014). There was increased CSPG expression at the site of injury, which peaked at 2 weeks post-TX and then gradually decreased to control levels by 10 weeks. In lamprey, both PTPσ and LAR mRNAs were expressed primarily in neurons whose regeneration capacity is poor (bad regenerators) in both control brains and brains of animals with SCI. Although ISH suggested that both PTPσ and LAR were upregulated after spinal cord TX, the effect was not quantitated at that time. The PTPσ mRNA-positive identified RS neurons often included M_2_ and M_3_ in the mesencephalon, and I_1_, I_2_, Mauthner (Mth), B_1_, B_3_, and B_4_ in the rhombencephalon. In the present study, PTPσ mRNA also appeared dramatically in some axons with poor−regenerative ability (including Mth, B_3_, and I_2_), consistent with the pattern of expression observed in the cell bodies in the brainstem. Of special interest, digestion of CSPGs with ChABC applied at the site of injury reduced the number of RS neurons expressing PTPσ mRNA, suggesting that the upregulation of PTPσ mRNA observed in RS neurons after SCI is due in part to the actions of elevated CSPGs. Although we cannot determine from the present study whether this is due to a direct effect of CSPGs on the injured axons, our findings are consistent with the recent studies. Lang et al. (2015) found that PTPσ becomes concentrated in dystrophic stabilized growth cones and LAR has similar elevation pattern. They also observed a large concentration of PTPσ in the lesion penumbra following SCI (Lang et al., 2015). Moreover, there is a report suggesting that CSPGs may upregulate PTPσ mRNA and protein levels in neural stem cells *in vitro* (Zhong et al., 2019). Thus, the ChABC digestion of CSPGs affects PTPσ expression in lamprey RS neurons, which may reflect a direct action on the injured axon and a retrograde signal to the neuronal perikaryon.

### ChABC Activates Akt

PTEN knockout promotes potent CNS axon regeneration after injury (Park et al., 2008; Liu et al., 2010; Du et al., 2015), and the signaling molecules downstream of PTEN that mediate this effect have been studied extensively (Liu et al., 2011). It has been reported that Akt activation can promote optic nerve axon regeneration and survival of RGC (Yang et al., 2014; Guo et al., 2016). GSK3β plays an indispensable role in mediating Akt-induced axon regeneration. Deletion or inactivation of GSK3β promotes axon regeneration independently of the mTORC1 pathway, whereas constitutive activation of GSK3β reduces Akt-induced axon regeneration. eIF2B_ε_ has been identified as a novel downstream effector of GSK3β and inactivation of eIF2B_ε_ reduces both GSK3β and Akt-mediated effects on axon regeneration. Constitutive activation of eIF2B_ε_ is sufficient to promote axon regeneration, which reveals a key role of the Akt-GSK3β-eIF2B_ε_ signaling module in regulating axon regeneration in the adult mammalian CNS (Guo et al., 2016). Akt activation is sufficient to promote optic nerve regeneration, but the regeneration is not as robust as that with PTEN deletion (Yang et al., 2014). Akt plays similar roles in the regeneration of lamprey CNS axons after SCI. In the present study, ChABC treatment greatly promoted axonal regeneration at 10 weeks after SCI. This was accompanied by widespread enhancement of Akt activation (pAkt-308) in the brain. IHC in individual identified RS neurons confirmed the increase in Akt phosphorylation. Taken together, these findings support the idea that enhanced activation of Akt is involved in the axon regeneration induced by ChABC treatment after SCI in lamprey.

On the other hand, Akt is also a critical pro-survival molecule in stressed cells. Previous studies showed that Akt can be phosphorylated by phosphoinositide 3-kinase (PI3K), and thereby protect tumor cells from death (Mayer and Arteaga, 2016). PI3K/Akt activation signals damage in neural tissues after SCI (Du et al., 2015; Wang et al., 2017; Qi et al., 2019). Rapamycin treatment can activate Akt *via* phosphorylation (O’Reilly et al., 2006; Wullschleger et al., 2006), which has been reported to suppress apoptosis in several models of ischemia reperfusion injury (Lee et al., 2004; Carloni et al., 2009). Interestingly, we found that ChABC treatment protects identified RS neurons from undergoing apoptosis at 2 and 11 weeks post-TX. The activation of Akt after ChABC treatment is consistent with the observed effect on neuronal survival. We conclude that Akt activation contributes to the beneficial effect of ChABC treatment in protecting the RS neurons from undergoing retrograde apoptosis after SCI. A report on axon regeneration after small peptide-induced inhibition of PTPσ indicated that extracellular regulated kinases (Erks) also are involved in regeneration of peripheral axons (Yao et al., 2019). Another group of researchers specifically activated the ERK and Akt signaling pathways and performed a comprehensive study of neural regeneration in both PNS and CNS neurons in live Drosophila (Wang et al., 2020). They found that both ERK and Akt activations enhanced axon regeneration in the sensory neurons in Drosophila larvae (Wang et al., 2020). These reports strongly suggest that Erk might be involved in the downstream signaling pathway after ChABC treatment. We have also examined the roles of Erk in lamprey SCI model and explored the expression pattern after SCI in retrograde signaling (Jin et al., 2020). The specific role played by Erk after ChABC treatment in lamprey CNS is still unclear and under investigation.

## Data Availability Statement

The original contributions presented in the study are included in the article/supplementary material, further inquiries can be directed to the corresponding author/s.

## Ethics Statement

The animal study was reviewed and approved by the Temple University Institutional Animal Care and Use Committee.

## Author Contributions

JH did the immunohistochemistry and FLICA staining. GZ prepared the *in situ* hybridization probes. WR and JH performed the *in situ* hybridization and acquired the images. L-QJ and JH and carried out the western blotting. JH and MS designed the experiments, carried out the data analysis, and drafted and prepared the manuscript. SL contributed to the experimental design and suggested specific experiments. All authors had full access to all the data in the study and take full responsibility for the integrity of the data and accuracy of the data analysis.

## Conflict of Interest

The authors declare that the research was conducted in the absence of any commercial or financial relationships that could be construed as a potential conflict of interest.
